# Positron emission tomography and computed tomography angiography for the diagnosis of giant cell arteritis: A real-life prospective study

**DOI:** 10.1097/MD.0000000000004146

**Published:** 2016-07-29

**Authors:** Delphine Lariviere, Khadija Benali, Baptiste Coustet, Nicoletta Pasi, Fabien Hyafil, Isabelle Klein, Maria Chauchard, Jean-François Alexandra, Tiphaine Goulenok, Antoine Dossier, Philippe Dieude, Thomas Papo, Karim Sacre

**Affiliations:** a Département de Médecine Interne; b Département de Médecine Nucléaire; c Département de Rhumatologie; d Département de Radiologie, Hôpital Bichat, Université Paris Diderot, PRES Sorbonne Paris Cité; e Département de Médecine Interne, Hôpital Saint Antoine, Université Pierre et Marie Curie, Assistance Publique Hôpitaux de Paris; f INSERM U1149; g Département Hospitalo-Universitaire FIRE (Fibrosis, Inflammation and Remodelling in Renal and Respiratory Diseases), Université Paris Diderot, PRES Sorbonne Paris Cité, Paris, France.

**Keywords:** 18F-fluoro-deoxyglucose positron emission tomography scan, case-control study, computed tomography angiography, diagnosis, giant cell arteritis

## Abstract

The use of 18F-fluoro-deoxyglucose positron emission tomography scan (FDG-PET) and computed tomography angiography (CTA) to improve accuracy of diagnosis of giant cell arteritis (GCA) is a very important clinical need. We aimed to compare the diagnostic performance of FDG-PET and CTA in patients with GCA.

FDG-PET and CTA were acquired in all consecutive patients suspected for GCA. Results of FDG-PET and CTA were compared with the final diagnosis based on clinical judgment, temporal artery biopsy (TAB) findings, and ACR criteria. Sensitivity, specificity, and positive and negative predictive values (PPV, NPV) were calculated for each method.

Twenty-four patients suspected for GCA were included. Fifteen (62.5%) were ultimately diagnosed as having GCA. Among them, all fulfilled ACR criteria and 6 had biopsy-proven GCA. Strong FDG uptake in large vessels was found in 10 patients who all had GCA. Mean maximal standard uptake values (SUVmax) per patient measured at all the arterial territories were of 3.7 (range: 2.8–4.7). FDG uptake was negative in 14 patients including 9 and 5 patients without and with GCA, respectively. Mural thickening suggestive of aortitis or branch vessel arteritis was observed on CTA in 11 patients with and 2 patients without GCA. No mural thickening was observed in 11 patients including 7 patients without and 4 patients with GCA. Overall, sensitivity was 66.7% and 73.3%, specificity was 100% and 84.6%, NPV was 64.3% and 64.6%, and PPV was 100% and 84.6% of FDG-PET and CTA, respectively.

Both FDG-PET and CTA have a strong diagnostic yield for the diagnosis of GCA. FDG-PET appeared to have a higher PPV as compared to CTA and may be the preferred noninvasive technique to explore patients with suspected GCA.

## 1 Introduction

Diagnosis of giant cell arteritis (GCA) is still a challenge. The positive predictive value (PPV) of the American College of Rheumatology (ACR) GCA criteria^[1]^ is low in a clinical setting.^[2]^ Temporal artery biopsy (TAB) is the criterion standard but a negative finding on biopsy of the temporal artery cannot exclude GCA.^[3]^

Over the past recent years, 18F-fluoro-deoxyglucose positron emission tomography scan (FDG-PET) has emerged as an efficient tool for the detection of large-vessel involvement in patients with GCA. Positron emission tomography (FDG-PET) has been ascribed a sensitivity of 89.5% and a specificity of 97.7% for GCA diagnosis.^[4–7]^

Computed tomography angiography (CTA) may characterize mural thickening, inflammatory periaortic soft tissue changes, stenotic or aneurysmal lesions. Aortitis is shown by CTA in >50% of patients with new GCA diagnosis.^[4,8]^

To our knowledge, CTA and FDG-PET have not been compared face-to-face in previous studies. The results of these 2 imaging techniques were prospectively analyzed in all consecutive patients with suspected GCA.

## 2 Patients and methods

### 2.1 Patients

At our institution, a temporal artery biopsy (TAB) is systematically performed in all patients in whom GCA is suspected according to the Recommendations of the French Study Group for Large Vessel Vasculitis (GEFA).^[9]^ Between November 2013 and August 2015, all patients in whom a TAB was performed for suspected GCA were considered for participation in the study. Patients who had received glucocorticoid treatment for >7 days were excluded. The American College of Rheumatology (ACR) 1990 GCA criteria were used for classification.^[1]^ The diagnosis of GCA was established on an individual basis by experienced clinicians (DL, BC, JMC, FA, TG, AD, PD, TP, KS). Of note, clinical judgment included short-term outcome following corticosteroid therapy, such as rapid and dramatic improvement of clinical symptoms and normalization of the C-reactive protein (CRP) blood level. TAB was performed in all patients. In patients with negative TAB results, the clinical diagnosis was considered final if no diagnosis other than GCA was provided at the end of a follow-up period of >6 months. The study group included all patients with definite GCA. The control group included patients in whom the diagnosis of GCA was not confirmed according to the same criteria. The study was approved by the ethics committee (Institutional Review Board -IRB00006477- of Paris 7 University, AP-HP). All patients provided written informed consent.

### 2.2 FDG-PET-CT imaging protocol

After an overnight fast, 18-FDG was injected to patients at a dose of 4 Mega-Becquerels per kg. PET images were acquired 90 minutes after FDG injection using a combined PET-CT scanner (Discovery 690; GE Healthcare, CT, France). Low-dose CT (100 keV and 140 mA with current modulation system) without contrast enhancement was acquired for anatomic correlation and attenuation correction of the PET data. PET images were reconstructed using 3-dimensional time-of-flight ordered subset expectation maximization with and without attenuation correction and reoriented in axial, sagittal, and coronal slices (3 mm cross-section thickness and 256 × 256 matrix for a visual field of view of 60 cm). Reconstructed images were displayed on an Advantage Workstation (GE Healthcare) for visual analysis.

### 2.3 Image analysis of FDG-PET acquisitions

Assessment of PET data was carried out by 2 nuclear medicine specialists (KB and FH), who were blinded to clinical and pathological findings. The diagnosis of GCA was based on the presence of high FDG uptake in the vascular wall of aortic segments, supra-aortic branches, and iliac-femoral territories with visual analysis of PET images. In addition, the intensity of FDG uptake was quantified in vascular regions with high FDG uptake using target to background ratio, the ratio between maximal standard uptake values (SUVmax) in the vascular wall, and SUVmean of blood. Unequivocal masked evaluation could be guaranteed because of the study design and controls’ group selection.

### 2.4 CTA

In a consensus reading, 2 radiologists (NP and IK) analyzed the CT images. Both readers were blinded to the clinical and pathological findings. Mural thickening was evaluated using a 4-point ranking scale, as follows: 0 = no mural thickening, 1 = slight mural thickening, 2 = mural thickening, and 3 = long and strong circumferential mural thickening. The rankings of 0 and 1 were considered to represent a normal state. The rankings 2 and 3 were considered to represent signs of mural inflammation. Four aortic segments (ascending thoracic aorta, aortic arch, descending thoracic aorta, and abdominal aorta) and the main supra-aortic tributaries were evaluated.

### 2.5 Statistical analysis

Data were compared between groups using *χ*^2^ test (or Fisher) for categorical variables and Wilcoxon rank-sum test for continuous variables. Statistical significance was defined as *P* < 0.05. The diagnosis of GCA by CTA and PET was compared with the final diagnosis according to clinical judgment, TAB findings, and ACR criteria. Sensitivity and specificity as well as PPV and NPV were assessed. Statistical analysis was performed with GraphPad Prism 5.01 software.

## 3 Results

### 3.1 Clinical and laboratory findings of the patients

Twenty-nine consecutive patients in whom a TAB was performed because of suspected GCA were screened. Five patients had received glucocorticoid treatment for >7 days before imaging and were excluded. The remaining 24 were included. Fifteen (62.5%) were ultimately diagnosed as having GCA. TAB specimen microscopic analysis showed clear-cut vasculitis (i.e., mononuclear cells infiltrate in the medial layer) in 6 patients (40%). The ACR criteria were met in all 15 cases. The 9 patients in whom GCA diagnosis was not confirmed constituted the control group.

The frequency of cranial and systemic symptoms did not differ between GCA patients and controls. All GCA patients had an increased CRP blood level as compared to only 5 controls (100% vs. 55.5%, *P* = 0.012). No significant difference in age, sex, or cardiovascular risk factors was observed between patients and controls. Five patients with newly diagnosed GCA had been treated by oral prednisone at 0.7 to 1 mg/kg/day for a median of 4 (range: 1–7) days at the time of imaging.

Clinical and laboratory data of both groups are summarized in Table 1.

**Table 1:**
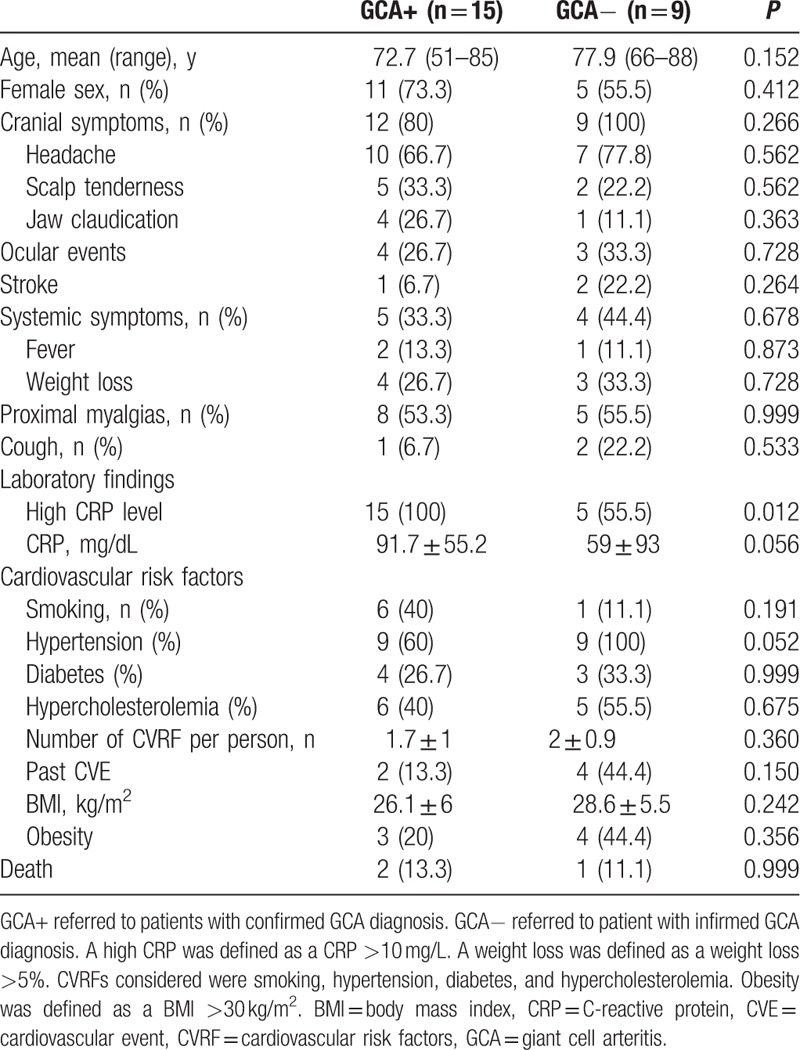
Patients’ characteristics.

### 3.2 FDG-PET of large vessels for GCA diagnosis

The 24 consecutive patients underwent FDG-PET imaging for large vessels study.

**Figure 1:**
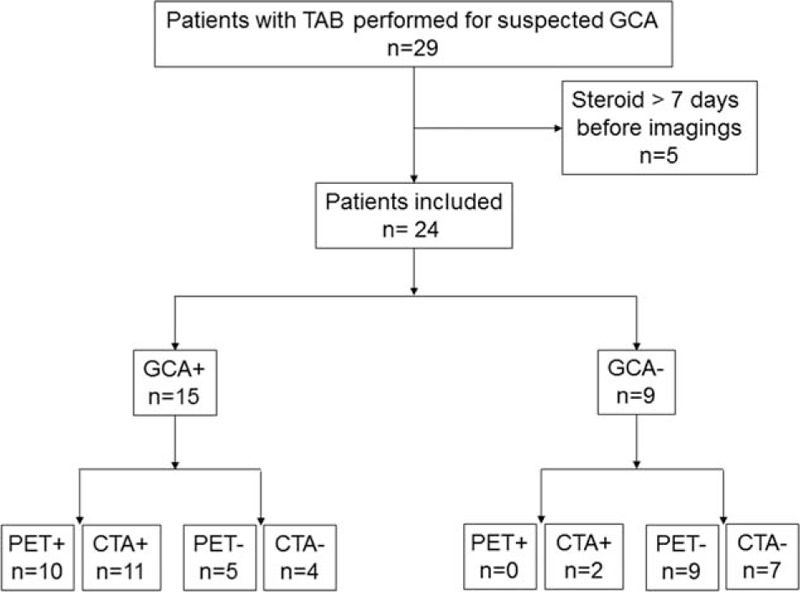
Studied patients. PET+ referred to FDG-PET showing strong FDG uptake in large vessels. PET− referred to FDG-PET with no FDG uptake in large vessels. CTA+ referred to CTA showing mural thickening suggestive of large vessels arteritis. CTA− referred to CTA showing no mural thickening. GCA+ referred to patients with confirmed GCA diagnosis. GCA− referred to patient with infirmed GCA diagnosis. CTA = computed tomography angiography, GCA = giant cell arteritis, PET = positron emission tomography combined with computed tomography, TAB = temporal artery biopsy.

**Figure 2:**
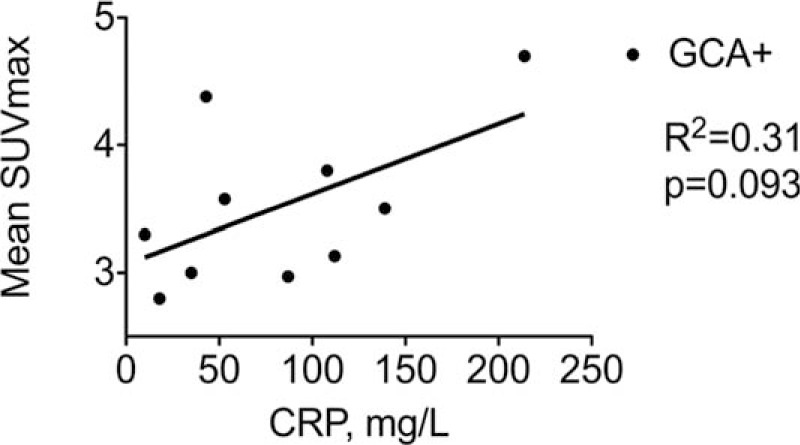
SUVmax and serum CRP level in patients with GCA. Mean SUV max referred to the mean SUVmax per patient measured at all the arterial territories in patients with GCA (GCA+, black circles). CRP referred to the maximum CRP value measured in serum per patient. CRP = C-reactive protein, GCA = giant cell arteritis, SUVmax = maximal standard uptake values.

Unequivocally strong linear FDG uptake in large vessels was found in 10 patients. All 10 patients had a definite diagnosis of GCA (Fig. 1). Of note, FDG uptake was observed despite steroid treatment in 3 patients. PET showed involvement of a mean of 6.9 ± 2.4 large arteries per patient including common carotid (n = 8, 80%), subclavian (n = 8), and vertebral arteries (n = 4), ascending thoracic aorta (n = 7), aortic arch (n = 8), descending thoracic aorta (n = 6), abdominal aorta (n = 4), and femoral arteries (n = 2). Supra-aortic vessels were involved in all cases. Mean of the maximum standardized uptake value (SUVmax) in the supra-aortic vessels, thoracic and abdominal aorta was of 3.6 (range: 2.5–6.1), 4.1 (range: 2.8–5.8), and 3.2 (range: 2.9–3.8), respectively. Mean SUVmax per patient measured at all the arterial territories was of 3.7 (range: 2.8–4.7) and tended to correlate with CRP blood level (Fig. 2). Of note, FDG-PET imaging studies revealed uptake in the shoulder and hip joints evocative of polymyalgia rheumatica in 3 patients. FDG uptake in the temporal artery was not seen.

FDG uptake was normal in 14 patients (Fig. 1). Nine patients had no GCA at the end of follow-up. Five patients had definite GCA and among them, 3 patients had a biopsy-proven GCA. Of note, 2 false-negative results were observed in patients already receiving steroids for 1 and 5 days at time of PET. No significant difference in clinical and laboratory data was found between GCA patients with positive and negative FDG-PET (data not shown).

Overall FDG uptake by large vessels yielded a sensitivity of 66.7% and a specificity of 100% for GCA diagnosis. The NPV and PPV of FDG-PET for the diagnosis of GCA were of 64.3% and 100%, respectively (Table 2).

**Table 2:**
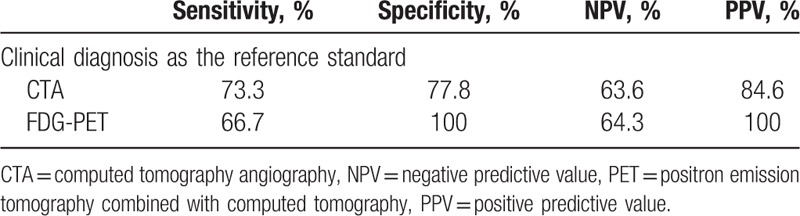
Diagnostic performance of CTA and FDG-PET imaging.

### 3.3 CTA of large vessel for GCA diagnosis

Mural thickening suggestive of aortitis or branch vessel arteritis was observed in 13 patients including 11 patients with GCA (true positive) and 2 patients without GCA diagnosis (false-positive) (Fig. 1). In GCA patients, CTA showed a mural thickening of a mean of 1.8 ± 0.9 large arteries per patient including supra-aortic vessels (n = 6), aortic arch (n = 1), descending thoracic aorta (n = 5), and abdominal aorta (n = 8). Of note, ascending thoracic aorta was never involved. No mural thickening was observed in 11 patients: 7 patients without GCA (true-negative) and 4 patients with GCA (false-negative). Two false-negative results were observed in patients already receiving steroids for 1 and 7 days at time of CTA.

CTA and PET lead to the same conclusion in 19 of 24 patients (79.2%). In GCA patients showing large vessels involvement according to CTA, mural thickening was associated with FDG uptake in 65% of cases (13/20 arterial segments involved). Conversely, FDG uptake in large vessels was associated with mural thickening in 37.1% in the same arterial segments.

Overall, CTA yielded a sensitivity of 73.3% and a specificity of 77.8% for GCA diagnosis. The PPV and NPV of CTA for the diagnosis of GCA were of 84.6% and 63.6%, respectively (Table 2).

## 4 Discussion

Our comparative study, conducted prospectively in consecutive patients suspected for GCA, shows a higher performance of FDG-PET as compared to CTA for noninvasive diagnosis of GCA.

The use of FDG-PET, CTA, MRI, and ultrasound techniques to improve accuracy of diagnosis of GCA is a very important clinical need. However, such studies are difficult to perform because establishing a “criterion standard” for the diagnosis of GCA is difficult (one of the goals being to improve upon that gold standard), and because selecting and evaluating negative controls is important.

The ACR criteria are not designed to be diagnostic criteria, but rather “classification criteria” and it is well known that these criteria perform poorly for diagnosis. It is also well-known that the TAB has inadequate sensitivity. In our study of patients highly suspected for GCA according to symptoms and inflammatory markers, the diagnosis of GCA was made according to clinical judgment, TAB findings, and ACR criteria. Without being an absolute criterion standard, we believe that such overall assessment based on expert clinician reflects the real-life practice.

In most controlled studies, controls are retrospectively selected age- and sex-matched patients without any features of GCA.^[5,7]^ On the contrary, our study was performed in “diagnostic conditions” and included consecutive patients with either confirmed or suspected (but with eventually another diagnosis made) GCA. Such design in real life allowed a better assessment of FDG-PET and CTA validity for GCA diagnosis.

GCA is now considered as the most common form of aortitis, with long segment thickening and smooth distal tapering, especially in the descending aorta and subclavian arteries.^[8–12]^ CTA allows precise imaging for aortitis and branch vessel arteritis. Using CTA, the prevalence of aortitis in GCA stands between 20% and 65%.^[13,14]^ CTA provides data on luminal anatomy—such as ectasia, dilation, stenosis, or occlusion—and detailed vessel wall characterization, including extent of mural thickening. Moreover, CTA is independent of the observer, shows high reproducibility, and allows high-quality documentation, helpful for comparability and sequential studies.

Recent meta-analysis on FDG-PET used as a diagnosis tool for GCA showed that the pooled sensibility and specificity of FDG-PET to detect the large-vessel vascular inflammation was 89.5% and 97.7%, respectively.^[5,7]^ The main specificity challenge of FDG-PET in older patients with GCA is to distinguish between true aortitis and atheroma—FDG uptake being more focal and restricted to aorta in atheroma—whereas sensitivity may be impeded because of steroid therapy that lowers FDG uptake. In our study, FDG-PET analysis was based on both visual assessment (i.e., strong diffuse linear pattern of FDG uptake in the aorta and its main branches) and quantitative scoring using blood uptake as a reference. Cases with strong or no strong FDG uptake were respectively considered unequivocally positive or negative. Moreover, in all instances, the FDG-PET reader was blinded to the clinical data and to the findings of the TAB, which was performed after the imaging procedures in all cases. FDG-PET yielded false-negative results in 2 patients that had received steroids for 1 and 5 days before undergoing imaging suggesting that even a short course of therapy may reduce the diagnostic accuracy of FDG-PET. Interestingly, FDG-PET may also be useful in diagnosing relapse, evaluating steroid refractory disease or predicting risk of later aortic dilation.^[8,15]^ In good agreement with previous report, we observed that FDG uptake assessed by the mean SUVmax at all the arterial territories tended to correlate with CRP blood level.^[6,16]^

Our study has obvious limitations. It is a monocentric study including a small number of patients. Larger trials are warranted to gain more reliable data for statistical purposes.

In conclusion, both FDG-PET and CTA are useful tools for GCA diagnosis. From our results, FDG-PET appears to have a higher PPV as compared to CTA and should stand as the best noninvasive technique for GCA diagnosis. Accordingly, a “no-biopsy strategy” could be assessed in patients suspected for GCA with a strong FDG uptake in large vessels.
